# Exploring restaurant and customer needs, barriers, interests, and food choices induced by the COVID-19 pandemic in Tarragona Province (Catalonia, Spain): A cross-sectional study

**DOI:** 10.3389/fpubh.2023.1137512

**Published:** 2023-04-11

**Authors:** Maria Besora-Moreno, Judit Queral, Silvia Torres, Elisabet Llauradó, Lucia Tarro, Rosa Solà

**Affiliations:** ^1^Functional Nutrition, Oxidation, and Cardiovascular Diseases Group (NFOC-Salut), Facultat de Medicina i Ciències de la Salut, Universitat Rovira i Virgili, Reus, Spain; ^2^Institut d’Investigació Sanitària Pere Virgili (IISPV), Hospital Universitari San Joan de Reus, Reus, Spain; ^3^Gasol Foundation, Sant Boi de Llobregat, Spain; ^4^Hospital Universitari Sant Joan de Reus, Reus, Spain

**Keywords:** COVID-19, restaurant, customer, needs, barriers, mediterranean-offer

## Abstract

**Background:**

COVID-19 has harmed restaurants, but customer preferences remain unknown. This study aims to determine the needs, barriers, interests, and food choice changes in restaurants and customers before and during the COVID-19 pandemic in Tarragona Province (Spain).

**Methods:**

An observational cross-sectional study conducted in spring 2021 collected Mediterranean offerings, food safety, and hygiene information about the pandemic through online surveys and focus group interviews with restaurateurs and customers about the changes in their needs and new barriers.

**Results:**

Fifty-one restaurateurs (44 survey, 7 focus group) and 138 customers (132 survey, 6 focus group) were included. In relation to the economic, emotional, and uncertainty restaurateurs’ barriers detected, they implemented measures to tackle it: buy less and more often, reduce restaurant staff and reduce the restaurants offer, among others. Some customers reported changes in their restaurant orders, specifically increasing their takeaway orders. The Mediterranean diet offer (AMed criteria) remained without noticeable changes in any of the criteria. After lockdown, compared to before lockdown, restaurateurs increased their takeaway offerings by 34.1% (*p* < 0.001) and their use of digital menus by 27.3% (*p* < 0.001) because of customer demand. The use of local products in the menus remained high. The cleaning and disinfection tasks increased by 21.1% (*p* = 0.022), and the use of hydroalcoholic solutions increased by 13.7% (*p* = 0.031).

**Conclusion:**

In restaurants, the first COVID-19 lockdown increased takeaway orders, sanitation, and digital communication. This study provides valuable information for adapting gastronomic offerings during challenging situations.

## 1. Introduction

Recently, a worldwide pandemic caused by the SARS-CoV-2 virus broke out, various social and mobility restrictions have since been implemented (1), and Spain specifically was hit hard by the COVID-19 pandemic (2).

Before the COVID-19 pandemic, there had been a growing trend toward consuming food outside the home (3, 4), which was motivated by socioeconomic changes such as the increased involvement of women in work and a lack of time at home (5). This trend has been reduced due to the significant negative impact of the pandemic on the restaurant sector and the closure of restaurants (1, 6). An analytical *Google Trends* study showed that during the first months of 2020, which included the pandemic lockdown period, people’s interest in restaurants dropped substantially worldwide, while interest in food security and takeaway food increased (7) due to the desire to reduce the risk of exposure to the virus, which led to online food purchases (8).

Although the evidence from Spain and Brazil suggests that SARS-CoV-2 is not transmitted through food (9, 10) and that it spreads primarily through small respiratory droplets in enclosed spaces, in the United States, the restaurant sector experienced a significant revenue shortfall caused by a sharp decline in customer demand and temporary interruptions of processes (2).

Moreover, during the first wave of the COVID-19 pandemic, people were in lockdown, and the changes in their restaurant food choice preferences remain unknown. Focusing on nutritional habits, an observational study from Poland suggests that during the pandemic, most of the population did not change their diet (11). However, 20% of people improved their eating habits with healthier eating, while another 20% of respondents worsened their eating habits (11). In addition, during the first COVID-19 pandemic lockdown, due to the increase in online work and the restrictions implemented, home delivery and takeaway orders increased, while the type of dishes requested did not vary relative to before the pandemic (11). Knowledge of the criteria used by customers when selecting a restaurant is critical for understanding food consumption trends (3). Therefore, to improve the ability of restaurant owners to respond adequately to customers, they must understand the behaviors of and factors influencing the decisions of consumers in the restaurant sector (3).

Despite the current negative circumstances arising from the COVID-19 pandemic, these circumstances have provided an opportunity to improve the resilience of the sector, defined as the ability to plan and prepare to adapt and recover from adverse situations (10). Two studies from Brazil and China highlighted that a common way for restaurants to adapt and reduce the impact of the COVID-19 pandemic was to increase their takeaway and food delivery offerings (10, 12). Thus, restaurants continued to offer their services and support at least some of their workers (10, 12).

To our knowledge, the present study is the first to attempt to determine the real impact of the COVID-19 pandemic on restaurants at a regional level.

Our hypothesis is that the needs, barriers, interests, and food choices of restaurants and customers differ before and during the current COVID-19 pandemic.

The main objective of this study was to determine the changes in restaurant and customer needs, barriers, interests, and food choices before and during the current COVID-19 pandemic in Tarragona Province (Spain). The specific objectives of this study were to identify the changes that restaurants and customers underwent before and during the current COVID-19 pandemic, specifically those related to food safety and food hygiene.

## 2. Materials and methods

### 2.1. Design and study population

The present study is an observational cross-sectional study that was conducted from April to June 2021 in Tarragona Province, Catalonia (Spain).

The study population was the owners of restaurants located in Tarragona Province and customers. The recruitment of restaurants was carried out by email and telephone calls, while customer recruitment was carried out by social networks. In addition, to ensure a proportionate number of restaurants was selected in each area, the number of inhabitants in the 10 counties of Tarragona Province was taken into consideration (13). Therefore, more restaurants were sampled from counties with more inhabitants (14).

In the restaurant sector, there were some restrictions such as a limit of customers inside the restaurant, a limit of the customers per table, 2 m between tables and limitations on the opening hours, and perimeter lockdown with their important consequences of society mobility. To determine the changes in customer and restaurant needs, barriers, interests, and food choices experienced by restaurateurs suffering under the circumstances of the COVID-19 pandemic, the following approaches were used:

- An online survey that referred to the period before and during the current COVID-19 pandemic situation was conducted with the two study populations, restaurateurs and customers, to obtain quantitative information on the changes of interest. This type of quantitative approach identifies the total changes made by restaurateurs and customers.
- Focus groups, formatted as a structured debate between a group of participants who could freely contribute their opinions and directed by a moderator (15) and in which questions were asked that referred to the period before and during the current COVID-19 pandemic situation, were arranged to provide qualitative information. The focus groups included members of the two populations, restaurateurs and customers, to identify their needs, barriers and changes implemented from the COVID-19 pandemic. This type of qualitative approach provides the reason for the changes in the needs of and barriers experienced by restaurateurs and customers.

This cross-sectional study followed the guidelines in the Strengthening the Reporting of Observational Studies in Epidemiology (STROBE) Statement (16) (Supplementary material 1) and the Consolidated Criteria for Reporting Qualitative Research (COREQ; Supplementary material 2) (17). The protocol for this study was approved by the Ethics Committee of the Pere Virgili Institute (ref. 056/2021). All participants provided signed informed consent before their participation.

### 2.2. Inclusion and exclusion criteria

For the restaurants, the inclusion criteria were as follows: (1) being a restaurant, hotel with a restaurant or camping ground including restaurant; (2) being located within Tarragona Province (Catalonia, Spain); (3) having a menu that includes dishes with local and seasonal foods; and (4) having signed the informed consent before the study. The restaurant exclusion criteria were as follows: (1) being a fast-food or ethnic restaurant; (2) offering only one type of product; and (3) belonging to a food chain. The restaurateurs of the included restaurants answered the survey and participated in the focus group. The restaurateurs were recruited through emails and phone calls.

Regarding restaurant customers, the inclusion criteria were as follows: (1) being over 18 years of age and a customer of restaurants located in Tarragona Province (Catalonia, Spain); (2) signing the informed consent before the study; (3) accepting the data protection conditions; and (4) accepting the privacy policy. The exclusion criterion for customers was failing to meet at least one inclusion criterion. Customer recruitment was performed through social networks.

### 2.3. Outcomes

The principal outcome was Mediterranean food offerings as assessed by the criteria for obtaining a Mediterranean Diet (AMed) accreditation (18). The AMed accreditation for restaurants that guarantees the offering of a menu based on the Mediterranean diet is led by the General Directorate of Public Health in the Department of Health of the Government of Catalonia.

**Figure 1:**
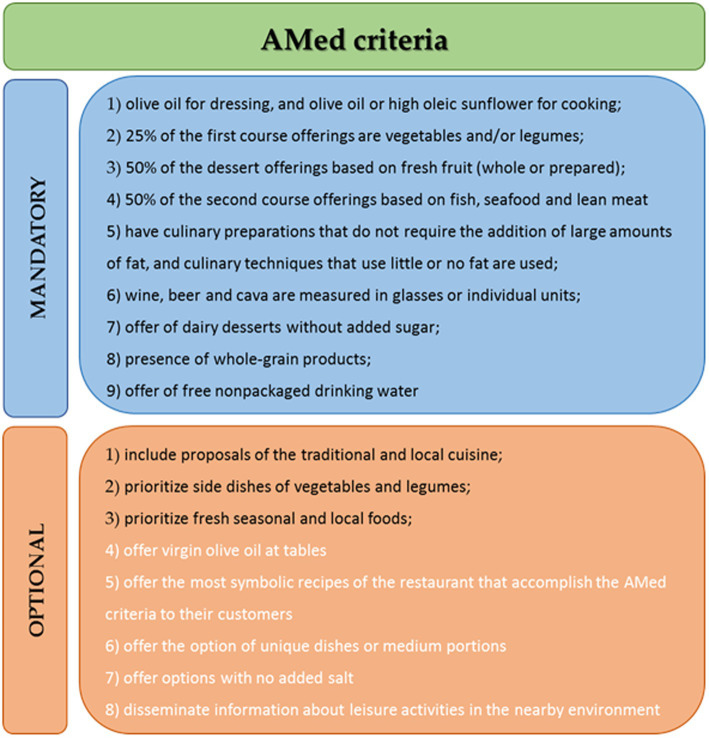
AMed criteria. The black quotes were the included in the analysis, and the white quotes were excluded.

The AMed accreditation contains a total of 17 criteria, 9 mandatory and 8 optional criteria. In the present study, to assess Mediterranean food offerings, 12 of the 17 AMed criteria were used, all 9 mandatory criteria and 3 of the 8 optional criteria, as shown in Figure 1 (available at www.amed.cat, accessed on 20 February 2021). Twelve of the 17 criteria were evaluated, but for the purpose of achieving the primary outcome, only the nine mandatory criteria were considered: (1) olive oil is used in dressings, and olive oil or high oleic sunflower are used for cooking; (2) 25% of the first course offerings are vegetables and/or legumes; (3) whole-grain products are included; (4) 50% of the second course offerings are based on fish, seafood, or lean meat; (5) 50% of the dessert offerings are based on fresh fruit (whole or prepared); (6) dairy desserts without added sugar are offered; (7) free non-packaged drinking water is offered; (8) wine, beer, and cava are measured in glasses or individual units; and (9) culinary preparations that do not require the addition of large amounts of fat and culinary techniques that use little or no fat are used. The 3 optional criteria analyzed were (1) including proposals of traditional and local cuisine, (2) prioritizing side dishes of vegetables and legumes, and (3) prioritizing fresh seasonal and local foods. The reason to select only 3 to 8 optional criteria was to prioritize the most adequate criterion to show changes in food restaurant offerings. Moreover, the criteria related to offering virgin olive oil in restaurant tables were excluded because the government had limited it for the COVID-19 pandemic situation.

The outcomes related to the AMed criteria before and during the current COVID-19 pandemic were reported at the same time.

The secondary outcomes from before and during the current COVID-19 pandemic included in this study were as follows:

Restaurants:

- Provision of services and food safety and hygiene (19).
- Barriers and needs: supplying healthy foods, using local products, addressing food allergies, and maintaining food hygiene and safety.

Customers:

- Consumption habits, evaluation of the supply of dishes corresponding to the Mediterranean diet in the restaurant, and restaurant selection criteria.
- Barriers and needs: restaurant visits, restaurant choice, perceptions of healthy food offerings, and management of food allergens.

### 2.4. Data collection

Data were collected through online surveys and focus groups with both restaurateurs and customers. Two different surveys were designed, one for restaurateurs and one for customers, which were answered during April–May 2021 and included questions related to the situation before and during the COVID-19 pandemic.

#### 2.4.1. Surveys

All surveys (restaurateur and customer) were answered in online format.

The restaurateur survey was not anonymous; however, the data were treated anonymously using a code that was known only to one researcher. This survey was created by the authors and has 3 separate sections:

- General information about the restaurant: name of the restaurant; type of establishment (restaurant, hotel, or camping ground); property characteristics (location, dimensions, capacity, terrace layout, type of ventilation, number of workers); last year’s assessment (billing, reductions in personnel and temporary filing of occupation regulation applications, reductions in orders to suppliers, increases in expenses due to additional measures, financial aid); services offered before and after the COVID-19 pandemic (local product dishes, dishes without food allergens, vegetarian/vegan dishes, payment and order methods, menu formats, type of dishes consumed in the restaurant and as takeaway). This section included 23 questions that allowed us to describe the type of restaurant and its characteristics, while the last questions of this section responded to the changes made by restaurants during and after the COVID-pandemic. Questions were designed by the authors following the rules imposed by the government to determine the main changes made by restaurants;
- The 12 selected Amed criteria (18) as “yes” or “no” answers, explained above;
- Hygiene and food safety issues as “yes” or “no” answers. This section referred to the 4 key points of hygiene and food safety of restaurants: cross-contamination, processes, cleaning and disinfection, and management. The questions of this section were based on “Guide to Good Practices for the Hygiene of Restaurants” (19), published by the Government of Catalonia and officially recognized by the competent authorities of Catalonia in matters of food safety. The customer survey was completely anonymous, and there was no question about personal information; therefore, it was not possible to identify the person responding to the survey. This survey had two different sections:
- Sociodemographic data. This section included 5 questions: sex, age, location, food allergy, or food intolerance, and whether customers had any difficulty finding restaurants with free allergen options.
- Consumption habits, interests, and food choices questions were designed by the authors by adapting questions from the findings of this publication (11) to our local environment. This section included 20 questions referring to food allergy diagnoses and suitable restaurants; food consumption behaviors: type of restaurants visited frequently; location of restaurants; frequency of restaurant visits or takeaway food orders; reasons for decreases in frequency; knowledge of the AMed accreditation; perceptions of each AMed criteria satisfied by the restaurant menu offerings (Mediterranean offerings) and 2 extra questions about food-allergen and vegetarian/vegan option perceptions by customers; and customer criteria for restaurant selection: advertising, price, variety and type of dishes offered, presentation, service, hygiene in the establishment, environment, and customer evaluations published by other customers.

#### 2.4.2. Focus groups

The focus groups were focused on (1) 6–12 restaurateurs (2 focus group sessions were conducted) and (2) 6–12 customers (1 focus group session was conducted) using the Teams online platform in line with the COVID-19 measures established by the competent local authorities. Each focus group session lasted 90 min. The attendees included one moderator, one study researcher and the restaurateur or customer participants.

The restaurateur and customer focus groups were organized around previously defined questions. Each focus group included the following six sections: (1) presentation of the participants; (2) presentation of the problem; (3) identification of barriers and needs; (4) food offerings (local products)/customer behavior regarding food; (5) food hygiene and safety; and (6) solutions from restaurateurs/solutions for restaurateurs provided by customers.

In addition, the moderator provided some examples to ensure participation and understanding of the questions. Furthermore, two interactive platforms, menti.com and Kahoot, were used to encourage interactions among the participants. At the end of each of the six sections, the moderator summarized the discussion, and the participants were requested to confirm the information. Moreover, the focus group was recorded in audio format, and the researcher assistant took notes on the spot. Then, the focus group recordings were transcribed for analysis, and a report was made available to participants to confirm what they had discussed during the session.

### 2.5. Sample size

The sample size calculation was based on the main variable: the 9 compulsory AMed criteria for restaurants (18). A standard deviation (SD) of ±1.6 was assumed, drawing upon the preliminary results of a cross-sectional study developed in the same area (20). A two-tailed alpha risk of 0.05, a two-tailed beta risk of 0.2, and a difference equal to or greater than 1 point in the AMed criteria score were considered for calculating the sample size of restaurants. A follow-up loss rate of 20% was estimated, considering the high number of restaurants that have closed due to the COVID-19 pandemic (13, 14). Additionally, the 10 counties were classified into 2 groups: 5 counties with fewer than 50,000 inhabitants and 5 counties with more than 50,000 inhabitants. Thus, 36 restaurants were needed considering the number of inhabitants per 10 counties, proportionally. The sample size was calculated using GRANMO software (21).

The restaurant customer sample size was not calculated because the principal variables, the 9 compulsory AMed criteria, are related to restaurants.

The focus group sample size was estimated based on the available evidence (22). The recommendation is that there should be between 6 and 8 participants in focus groups for noncommercial topics because more than 10 participants make it difficult for the focus group to develop. We over-recruited participants for the focus groups due to the possibility that someone did not attend. For this reason, our range of sample sizes was 6 to 12 participants in each focus group.

### 2.6. Data analysis

#### 2.6.1. Quantitative data

Categorical variables are reported as percentages, and continuous variables are reported as the mean ± SD. The McNemar test for categorical variables was used to compare the changes between the period before and the period during the pandemic. For comparison between restaurant vs. takeaway options during the same period of the pandemic^,^, the Chi-squared test was used. The significance level was fixed at *p* < 0.05. The statistical program SPSS version 27 was used (IBM Corp. Released 2020. IBM SPSS Statistics, Version 27.0. Armonk, NY: IBM Corp).

#### 2.6.2. Qualitative data

The focus groups were qualitatively analyzed using thematic analysis (23) to identify topics and themes, and the steps stated were followed to avoid researcher bias. The transcriptions of focus groups were reviewed, coded by open coding and then inductive methods, and discussed by two reviewers (MB-M, JQ). For interpretation of the focus group results, three different methods were used (24). First, the raw data, i.e., the exact words the participants said, were categorized according to the frequency with which something was said. Second, the descriptive information, i.e., a summary of the comments, was subsequently prepared by the analyst researcher who took notes during the focus group. Data were interpreted as raw data in summary form. Finally, the third method was the interpretive method, which consisted of the analyst researcher providing an interpretation of the data to help clarify the information obtained from the focus group.

## 3. Results

A total of 51 restaurateurs and 138 customers participated. Of the 51 restaurateurs, 44 participated in the survey and 7 in the focus group, whereas of the 138 customers, 132 participated in the survey and 6 in the focus group. Three online focus group sessions were conducted, 2 with restaurateurs and 1 with customers.

### 3.1. Results of the restaurant survey

#### 3.1.1. Characteristics of restaurants as described in the restaurant survey

Of the 44 restaurants represented by the restaurateurs, most were located in the two most inhabited counties of Tarragona Province in Catalonia, Tarragonès (31.8%, *n* = 14) and Baix Camp (25%, *n* = 11). The most frequent establishments were restaurants (88.6%, n = 39), followed by hotels (6.8%, *n* = 3) and camping grounds (4.5%, *n* = 2). Regarding the ventilation used by the restaurants, 65.9% (*n* = 29) used air conditioners, and 15.7% (*n* = 7) did not have ventilation.

Regarding marketing and communication media, most restaurants used social networks (90.9%, *n* = 40) both before and during the COVID-19 pandemic. Posters, leaflets, and brochures for menus were used before and during the pandemic.

As an important consequence of the first wave of the COVID-19 pandemic in restaurants, 31.8% (*n* = 14) of restaurateurs had to throw away food, and 43.2% (*n* = 19) gave food away (data not shown in tables).

Table 1 shows the main changes undergone by restaurants due to the COVID-19 pandemic. Takeaway food orders increased significantly by 34.1% (47.7% (*n* = 21) to 81.8% (*n* = 36); *p* < 0.001). Additionally, the percentage of dishes with local products was maintained at a high level, approximately 90% before and during the current COVID-19 pandemic.

**Table 1:** Restaurants’ characteristics changes due to COVID-19.

	Variables	Before COVID *n* = 44% (*n*)	Currently COVID^#^ *n* = 44% (n)	*p*-value^*^
Dishes offered	Dishes with local products	93.2 (41)	90.9 (40)	1
	Dishes without food allergens	88.4 (38)	86.0 (37)	1
	Vegetarian/vegan dishes	83.7 (36)	81.4 (35)	1
	Takeaway food	47.7 (21)	81.8 (36)	<0.001
Payment	Payment by credit card	100 (43)	95.3 (41)	_
	Payment with *bizum*	13.6 (6)	29.5 (13)	0.16
Menu format and demand	Menu in digital format	6.8 (3)	34.1 (28)	<0.001
	Use of ICT for the customer to place the order	4.5 (2)	13.6 (6)	0.219
	Use of ICT to write the customer’s choice	11.4 (5)	20.5 (9)	0.125
Sales	Sales	31.8 (14)	34.1 (15)	1
Changes in the tables	Cleaning dishes and cutlery at a temperature > 80°C	95.5 (42)	95.5 (42)	1
	Delivery of metal cutlery in sterilized bag	2.3 (1)	11.4 (5)	0.125
	Delivery of single-use cutlery	9.1 (4)	22.7 (10)	0.031
	Delivery of cloth napkins	52.3 (23)	38.6 (17)	0.031
	Delivery of single-use napkins	54.5 (24)	75 (33)	0.004
	Condiments available to customers as salt shakers and oil cruet	79.5 (35)	43.2 (19)	0.002
	Condiments available to customers in a single-dose format	9.1 (4)	70.5 (31)	<0.001

ICT: information and communication technology. ^#^Currently COVID-19 pandemic situation. ^*^McNemar test.

Regarding restaurant menu formats, the use of digital menus increased significantly by 27.3% (6.8% (*n* = 3) to 34.1% (*n* = 28); *p* < 0.001).

Additionally, the use of single-serving condiment packages increased by 61.4% (9.1% (*n* = 4) to 70.5% (*n* = 31); <0.001), and the use of single-use napkins increased by 20.5% (54.5% (*n* = 24) to 75% (*n* = 33); *p* = 0.004), whereas the use of cloth napkins, salthakers, and oil cruets decreased (*p* < 0.05), as shown in Table 1.

#### 3.1.2. AMed criteria results from the restaurant survey

According to the survey responses regarding the AMed criteria, of the 44 restaurants, only 4.5% (*n* = 2) had the AMed accreditation, 22.7% (*n* = 10) did not have the AMed accreditation but knew it, and 72.7% (*n* = 32) did not know the AMed accreditation (data not shown in tables).

Table 2 shows the results regarding the AMed criteria as reported by the restaurateurs. AMed criteria have not shown changes in any of the criteria studied before and after the COVID-19 pandemic. Focusing on the 9 mandatory AMed criteria, 100% (*n* = 44) of restaurants provided olive oil for dressing and olive oil or high oleic sunflower for cooking and provided seasonal and local products before the COVID-19 pandemic and continued to do so during the pandemic. In contrast, half or fewer of the restaurants satisfied the three mandatory criteria: (1) Offer dairy desserts without added sugar, (2) Prioritize side dishes of vegetables and legumes, and (3) Offer free non-packaged drinking water, and these results were unchanged during the pandemic, as shown in Table 2.

**Table 2:** Mandatory and optional AMed criteria fulfilled by the included restaurants reported by restaurateurs.

AMed criteria	Before COVID *n* = 44% (*n*)	Currently COVID^#^ *n* = 44% (*n*)	*p*-value^*^
**AMed mandatory criteria^1^**			
1. Olive oil for dressing, and olive oil or high oleic sunflower for cooking	100 (44)	97.7 (43)	---
2. 25% of the first course offerings are vegetables and/or legumes	95.5 (42)	93.2 (41)	1.000
3. 50% of the dessert offerings based on fresh fruit (whole or prepared)	95.5 (42)	93.2 (41)	1.000
4. 50% of the second course offerings based on fish, seafood and lean meat	93.2 (41)	90.9 (40)	1.000
5. Have culinary preparations that do not require the addition of large amounts of fat, and culinary techniques that use little or no fat are used	88.6 (39)	86.4 (38)	1.000
6. Wine, beer and cava are measured in glasses or individual units	86.4 (38)	90.9 (40)	0.500
7. Offer of dairy desserts without added sugar	54.4 (24)	56.8 (25)	1.000
8. Presence of whole-grain products	43.2 (19)	40.9 (18)	1.000
9. Offer of free non-packaged drinking water	25.0 (11)	29.5 (13)	0.500
Number of total AMed compulsory criteria fulfilled per restaurant (mean ± SD)^3^	6.8 ± 1.17	6.7 ± 1.47	
**AMed optional criteria^2^**			
10. Include proposals of the traditional and local cuisine	100 (44)	97.7 (43)	---
11. Prioritize side dishes of vegetables and legumes	93.2 (41)	93.2 (41)	1.000
12. Prioritize fresh seasonal and local foods	87.7 (43)	95.5 (42)	1.000

SD, Standard Deviation; AMed, Mediterranean Diet offer. 1: 9 compulsory AMed criteria; 2: 3 optional AMed criteria assessed from the 8 described in the Mediterranean Accreditation; 3: 12 total AMed criteria assessed from the total 17 AMed criteria. ^*^McNemar test. ^#^Currently COVID-19 pandemic situation.

#### 3.1.3. Hygiene and food safety results from the restaurant survey

As the supplementary table (Supplementary material 3) shows, the number of restaurants that cleaned the goods reception area frequently, specifically, the number of restaurants cleaning that area ≥ 2 times/day, increased by a significant 21.1% (72.1 (*n* = 31) to 93.2 (*n* = 41); *p* = 0.022). In addition, during the COVID-19 pandemic, the use of a hydroalcoholic solution, specifically for disinfecting cooking utensils, increased by a significant 13.7% from its level before the pandemic (6.3 (*n* = 3) to 20.5 (*n* = 9); *p* = 0.031). The other items, which remained unchanged, are described in Supplementary material 3.

### 3.2. Results of the restaurateur focus group

A total of 7 restaurateurs participated in the focus group session, which lasted 90 min. During the session, after introducing the participants and moderator, 5 topics were discussed.

#### 3.2.1. Problems due to COVID-19 in the restaurant sector

The restaurateurs were not satisfied with the way the authorities handled the COVID-19 situation in the restaurant sector. The restaurateurs believed that the measures imposed on this sector harmed employers, workers, and suppliers more than in other sectors where the measures were less stringent. (a) In addition, the lack of clarity in the messages issued by the administration and the short time that the restaurateurs had to adapt to the new measures, which were constantly changing, was a problem for them. (b) Moreover, some measures were difficult to implement, such as maintaining the required distance between customers (c).

- “There have been many people affected by the issue. It has been economic chaos for many people, not just for employers, but for workers who have stopped earning, for suppliers who have not been able to serve, and many other things.”
- “The measures have been too strong, but at the same time they have made us dizzy, because it was now yes, now no, now this way, now this other way, and so on. Many have fallen, others have endured as best we could, but it has been almost a year and a half where almost no one has been able to make a profit.”
- “The tables must be separated by 2 m. However, customers at the same table must be 1 m apart. The problem is that anyone has accomplished the measures because these are impossible to accomplish.”

#### 3.2.2. Barriers of restaurateurs

The main barrier was the restrictions imposed by the authorities, specifically the national and municipal lockdown, and the rapid changes in limitations meant that large amounts of food, especially fresh products, had to be given away.

**Figure 2:**
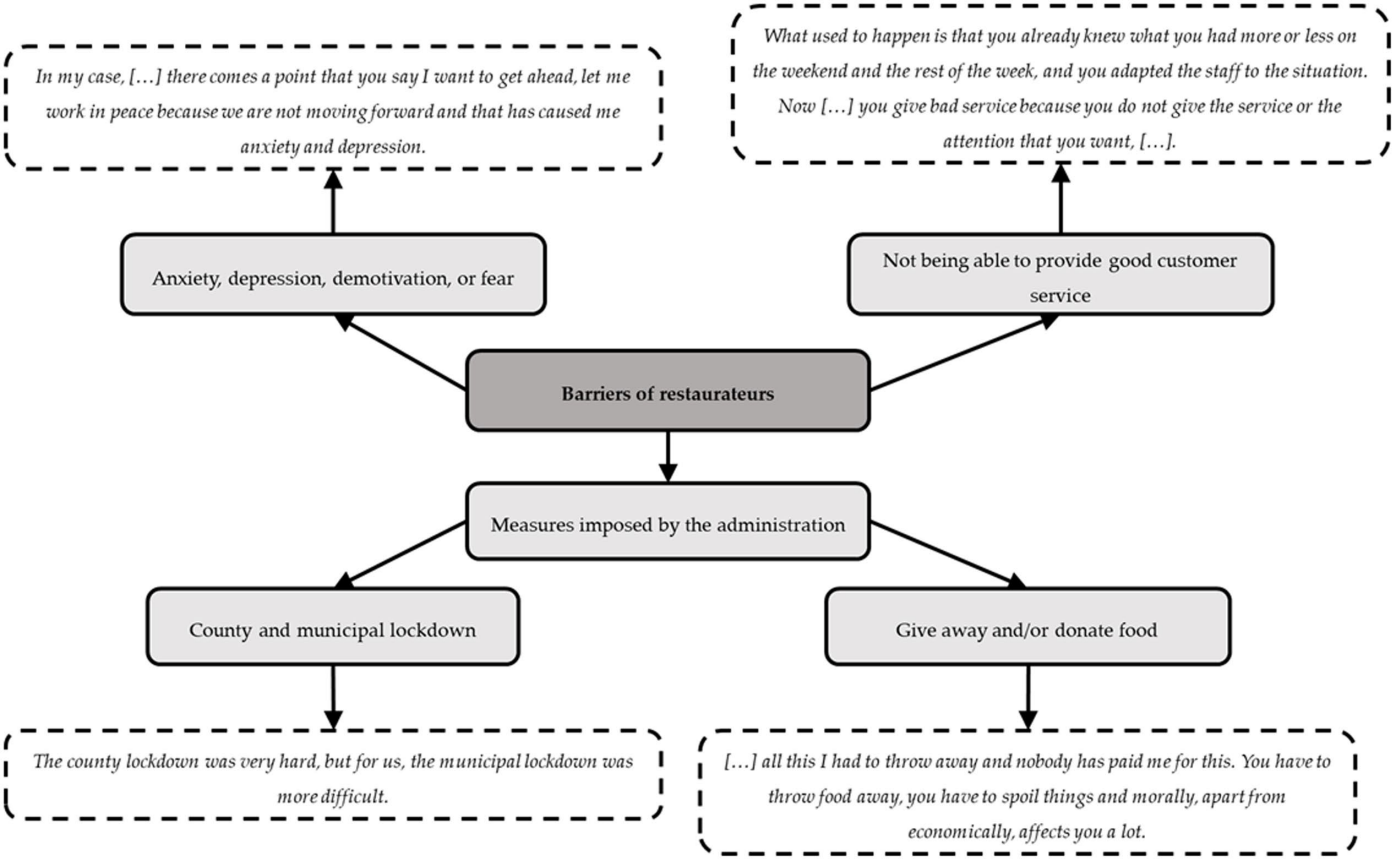
Barriers of restaurateurs.

In addition, other important barriers were (1) economic, as the number of customers significantly decreased and consequently the income of the restaurateurs; (2) emotional barriers, such as anxiety, depression, demotivation, or fear of being infected and having to close their restaurant and of not having an income; and (3) not being able to provide good customer services because the limitations placed on hours and the number of customers prevented them from predicting how many people they could hire or how much food they would need. These barriers are described in Figure 2 with some examples from the focus group transcriptions.

Due to the barriers mentioned above, the restaurateurs needed to increase their income and reduce their costs; for this reason, the restaurateurs implemented certain measures such as buying food daily instead of weekly or reducing the restaurant staff, eliminating daily menus and reducing the number of dishes and products supplied to provide better service (d)(e).

In addition, other measures included web page updates and providing menus in QR format. Other tactics included commercializing their menus in supermarkets, providing takeaway services, or delivering food to nearby towns. (f) However, the restaurateurs commented that it was easier to make fast food for takeaway orders than local dishes.

- “We cannot have a daily menu with only two people working. It has been necessary to reduce the staff, we have limited the supply, it is a lot of things. We are continuously making special offers to attract the people.”
- “What we have had to do is to buy daily. Before, I used to place orders twice a week and now we make a forecast for the next day, and especially with the fresh products, we buy them daily, precisely so that what has happened to us does not happen again.”
- “We had never offered takeaway food, and having to close, being in such a small town, it has been very good for us. I took the car and went through the Ribera d’Ebre and Terra Alta distributing, and that has helped us a lot, but in the long run, it is not an option that we value.”

#### 3.2.3. Food offered (local products)

The main restaurants provided healthy traditional and Mediterranean cuisine. The restaurants used local products, especially fruits, vegetables, and fish, and they did not have any problems obtaining products during the lockdown. (g) Although restaurateurs did not observe any changes in the demand for special dishes, they stated that more people are vegetarian or have food intolerances. All of them found that takeaway food was not a problem for these special cases (h).

- “We work with seasonal and km 0 products, so nothing has changed for us.”
- “I have not noticed a change; there are more and more people who have special diets. Some diets are voluntary, such as veganism or vegetarianism, or the thousand variants that there are, or forced diets, such as people who have various allergies. However, we have to offer gluten-free bread, have a supply that considers these people, if not you further reduce the number of people who can come to your establishment.”

#### 3.2.4. Food hygiene and safety

Regarding food hygiene and safety, all the restaurateurs agreed that they handled and worked with foods in the same way as before COVID-19. Focusing on food delivery, they did not use isothermal bags to deliver the food, and only two restaurateurs included some instructions about the handling and storage of the delivered food (i).

- “The temperature chain can be broken. On New Year’s Eve, we had more than 100 daily meals, all takeaway and delivery. In addition, everything had to be heated later. However, we always give daily meals with an instruction sheet that explains how each dish should be heated and presented.”

#### 3.2.5. Solutions from restaurateurs

The possible solutions ideated by the restaurateurs were to reinvent themselves, generate income from atypical sources, and reduce expenses (j)(k)(l).

- “Anyway, we have passed through it, we are enduring it, we are tired of enduring it, and the only solution is for it to end, for the restrictions to end and they let us work.”
- “It can help the people, that the COVID [lockdown] ends and that people come because the administration will not help with anything.”
- “It seems to me that we have to learn from each situation and adapt ourselves.”

### 3.3. Results of the customer survey

#### 3.3.1. Characteristics of customers as described in the customer survey

A total of 132 restaurant customers in Tarragona Province answered the survey, which focused on the differences in their choices before and during the current COVID-19 pandemic. Related to demographic characteristics, most were located in three counties of Tarragona Province in Catalonia, 59.1% (*n* = 78) in Baix Camp, 13.6% (*n* = 18) in Montsià, and 12.9% (*n* = 17) in Tarragonès. Of the 132 customers, 34.1% (*n* = 45) were men, and 65.9% (*n* = 87) were women, with a mean age of 41.7 ± 14.6 years.

#### 3.3.2. Characteristics of restaurant choices from the customer survey

Concerning the use of different digital formats to become aware of different options during the pandemic, specifically the use of marketing and communication media, the number of customers who selected restaurants through the use of social media significantly increased by 6% (37.9% (*n* = 50) to 43.9% (*n* = 58); *p* = 0.008), while the tendency to use websites for this purpose increased by 17.4% (41.7% (*n* = 55) to 59.1% (*n* = 78); *p* = 0.052). In addition, the number of customers with a preference for ordering from a digital menu increased by 57.5% since the start of the COVID-19 pandemic (20.5% (*n* = 27) to 78.0% (*n* = 103) *p* < 0.001). Regarding the method of payment, the number of customers who preferred to pay by credit card rather than by cash increased by 6.8% (84.1% (*n* = 111) to 90.9% (*n* = 120); *p* = 0.012).

Regarding the type of restaurant chosen by customers, after the start of the COVID-19 pandemic, the number of customers who preferred restaurants in rural areas over those in urban areas increased by 15.1% (22% (*n* = 29) to 37.1% (*n* = 49); *p* < 0.001). Moreover, the number of customers with a preference for terraces over indoor restaurant seating increased by 37.8% due to the pandemic (41.7% (*n* = 55) to 79.5% (*n* = 105); *p* < 0.001). In addition, the number of customers who reported eating outside of the home at least once a week decreased by 30.3% (74.2% (*n* = 98) to 43.9% (*n* = 58); *p* < 0.001), while the frequency of ordering food for takeaway out at least once a week increased by 12.9% after the start of the COVID-19 pandemic (6.8% (*n* = 9) to 19.7% (*n* = 26) *p* < 0.001).

#### 3.3.3. Customer preferences for in-restaurant or takeaway dishes from the customer survey

Focusing on customer preferences for in-restaurant or takeaway food, there were no significant changes between the period before and the period during the current COVID-19 pandemic, as Table 3 shows. However, customers preferred to order the following types of food as takeaway orders rather than in-restaurant orders in the current COVID-19 pandemic situation: themed dishes from different countries [restaurant: 41.7% (*n* = 55) vs. takeaway: 59.8% (n = 79); *p* < 0.001), fast food (restaurant: 10.6% (n = 14) vs. takeaway: 28.0% (*n* = 37); *p* < 0.001], and dishes suitable for those with allergies [restaurant: 3.9% (*n* = 5) vs. takeaway: 4.5% (*n* = 6); *p* < 0.001]. Instead, the dishes that customers chose more frequently for consumption in the restaurant than for takeaway included seasonal vegetable-based dishes [restaurant: 30.3% (*n* = 40) vs. takeaway: 6.1% (*n* = 8); *p* < 0.001] and vegetarian dishes [restaurant: 9.8% (*n* = 13) vs. takeaway: 6.8% (*n* = 9); *p* < 0.001].

**Table 3:** Preferences and comparison of dishes in the restaurant or takeaway.

Variables	Dishes chosen more often in the restaurant				Dishes chosen more often to takeaway or delivery				*p*-value restaurant vs. to takeaway currently COVID ^#$^
	Gender	Before COVID *n* = 132% (*n*)	Currently COVID^#^ *n* = 132% (*n*)	*p*-value^*^	Gender	Before COVID *n* = 132% (*n*)	Currently COVID^#^ *n* = 132% (*n*)	*p*-value^*^	
Traditional dishes	Total	81.8 (108)	80.3 (106)	0.625	Total	26.5 (35)	23.5 (31)	0.219	0.438
	Men	97.8 (44)	97.8 (44)	1	Men	33.3 (15)	31.1 (14)	1	1.000
	Women	73.6 (64)	71.3 (62)	0.625	Women	23.0 (20)	19.5 (17)	0.375	0.768
Dishes based on seasonal vegetables	Total	29.5 (39)	30.3 (40)	1	Total	6.1 (8)	6.1 (8)	1	<0.001
	Men	28.9 (13)	26.7 (12)	1	Men	6.7 (3)	4.4 (2)	1	0.467
	Women	29.9 (26)	32.2 (28)	0.5	Women	5.7 (5)	6.9 (6)	1	<0.001
Country themed food	Total	40.9 (54)	41.7 (55)	1	Total	56.1 (74)	59.8 (79)	0.125	<0.001
	Men	33.3 (15)	33.3 (15)	1	Men	55.6 (25)	55.6 (25)	1	0.027
	Women	44.8 (39)	46.0 (40)	1	Women	56.3 (49)	62.1 (54)	0.125	0.008
Vegetarian dishes	Total	9.8 (13)	9.8 (13)	1	Total	6.8 (9)	6.8 (9)	1	<0.001
	Men	4.4 (2)	4.4 (2)	1	Men	6.7 (3)	4.4 (2)	1	0.001
	Women	12.6 (11)	12.6 (11)	1	Women	6.9 (6)	8.0 (7)	1	<0.001
Dishes suitable for allergy sufferers	Total	5.3 (7)	3.8 (5)	0.5	Total	4.5 (6)	4.5 (6)	1	<0.001
	Men	2.2 (1)	2.2 (1)	1	Men	2.2 (1)	2.2 (1)	1	0.022
	Women	6.9 (6)	4.6 (4)	0.5	Women	5.7 (5)	5.7 (5)	1	<0.001
Fast food	Total	13.6 (18)	10.6 (14)	0.219	Total	30.3 (40)	28.0 (37)	0.508	<0.001
	Men	20.0 (9)	13.3 (6)	0.25	Men	31.1 (14)	26.7 (12)	0.625	0.035
	Women	10.3 (9)	9.2 (8)	1	Women	29.9 (26)	28.7 (25)	1	<0.001
Others	Total	0.8 (1)	1.5 (2)	1	Total	6.1 (8)	5.3 (7)	1	0.104
	Men	2.2 (1)	4.4 (2)	1	Men	8.9 (4)	8.9 (4)	1	0.172
	Women	0.0 (0)	0.0 (0)	-	Women	4.6 (4)	3.4 (3)	1	-

^*^McNemar test; ^$^Chi^2^ test. ^#^ Currently COVID-19 pandemic situation.

#### 3.3.4. Perceptions of each AMed criteria satisfied by the restaurant menu offerings (mediterranean offerings)

Table 4 describes the perceptions of each AMed criterion accomplished by the dishes offered. The use of olive oil for cooking and dressing was the most highly valued by 99.2% (*n* = 131) of customers, followed by the use of fresh, local, and seasonal foods, with 98.5% (*n* = 130) of customers valuing positively the accomplishment of this criterion, and the pro-vision of traditional dishes and/or typical local dishes, with 95.5% (*n* = 126) of customers valuing positively the accomplishment of this criterion. On the other hand, although the supply of vegetarian/vegan dishes, dishes without allergens, and whole grains was not highly valued by the restaurant customers, more than 50% positively valued the accomplishment of these criteria.

**Table 4:** Perceptions of each AMed criteria satisfied by the restaurant menu offerings (Mediterranean offerings).

Variables	Satisfied perceptions *n* = 132% (*n*)
Olive oil for dressing, and olive oil or high oleic sunflower for cooking	99.2 (131)
Prioritize fresh seasonal and local foods	98.5 (130)
Include proposals of the traditional and local cuisine	95.5 (126)
Prioritize side dishes of vegetables and legumes	89.4 (117)
25% of the first course offerings are vegetables and/or legumes	87.9 (116)
50% of the second course offerings based on fish, seafood, and lean meat	87.1 (115)
Offer of free non-packaged drinking water	85.6 (113)
50% of the dessert offerings based on fresh fruit (whole or prepared)	81.8 (108)
Have culinary preparations that do not require the addition of large amounts of Fat, and culinary techniques that use little, or no fat are used	81.8 (108)
Offer of dairy desserts without added sugar	77.3 (99)
Wine, beer, and cava are measured in glasses or individual units	68.2 (90)
*Include dishes without food allergens	67.4 (89)
Presence of whole-grain products	66.7 (88)
*Include dishes Vegetarian/vegan dishes	57.6 (76)

* Two extra questions regarding food-allergens and vegetarian/vegan options were included.

### 3.4. Results of the customer focus group

A total of 6 customers participated in the focus group, which lasted 90 min. During the focus group, after introducing the participants and moderators, 5 topics were discussed.

#### 3.4.1. Problems due to COVID-19 in the restaurant sector

The feelings that customers had during the pandemic were the loss of and longing for social relationships and freedom, uncertainty, fear of contracting and spreading the disease, insecurity when going to public places such as supermarkets and restaurants, and anxiety about not knowing when the situation would end. (a) Despite these feelings, they agreed that the restaurant sector was severely affected, especially economically, by the harsh restrictions (b).

- “I would also say that there has been a lot of social discomfort. On the one hand, you saw many people who were very afraid to go into public places, go to the supermarket, go to any bar, of meeting people for fear of contracting the disease, and on the other hand, you also saw people who were angry with the curfew due to the new schedules, which changed from time to time.”
- “We are a country in which we spend a lot of time on the streets because it is sunny, and we have a lot of daylight hours. Many times, we meet in a bar with friends and all these economic dynamics have perhaps been little contemplated, there was also all the pressure that for the first time we were all threatened by an unknown enemy. Therefore, mistrust has increased and this has led to very drastic measures.”

#### 3.4.2. Barriers of customers

**Figure 3:**
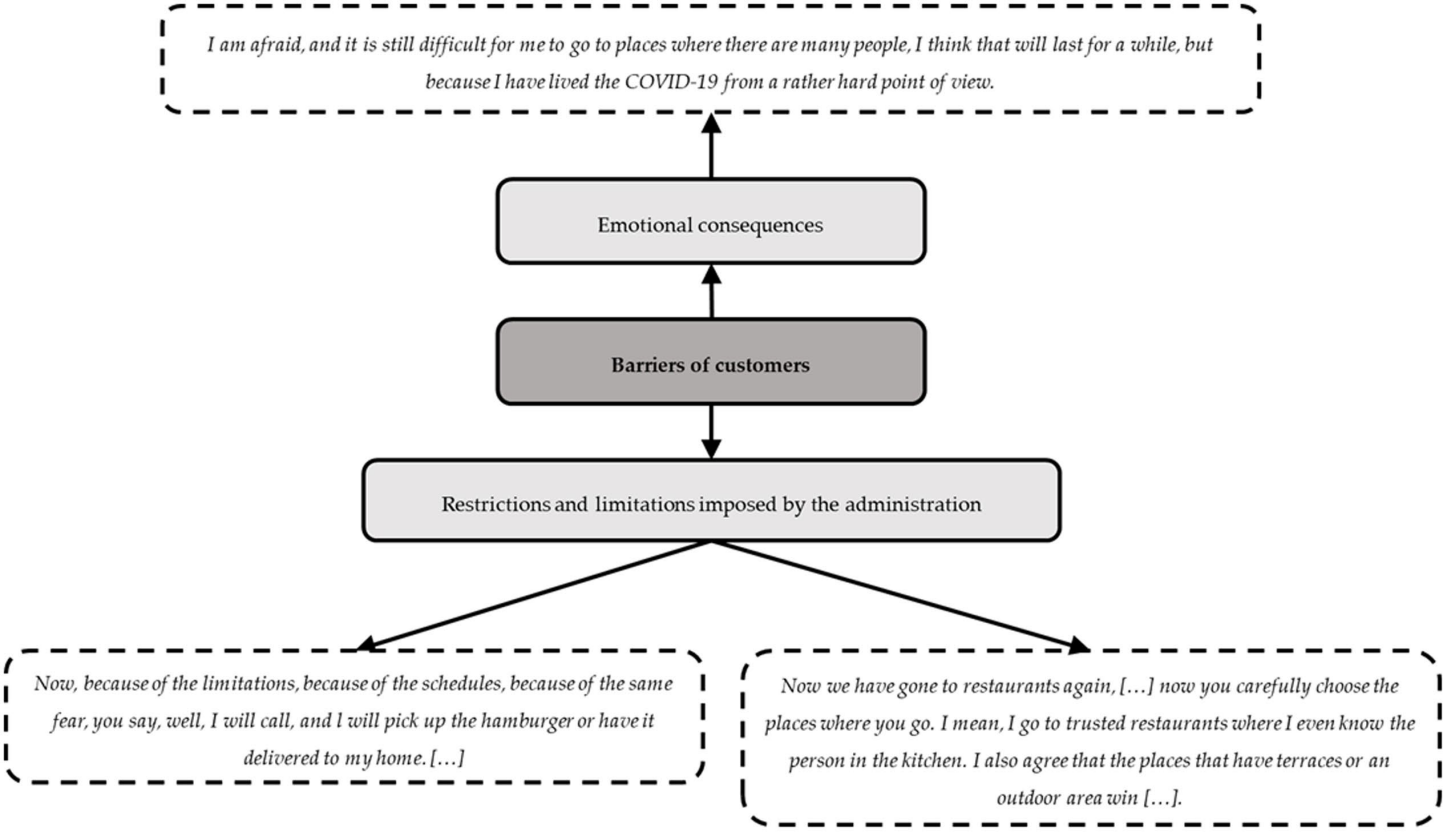
Barriers of customers.

The customers stated that they enjoy going to restaurants not only to eat but also for the experience, i.e., meeting other people, not needing to cook, and disconnecting from responsibilities. However, all this was interrupted by the COVID-19 pandemic. In this context, the main barriers that the focus group participants faced as customers were the restrictions and limitations; consequently, they preferred ordering takeaway food and being at home, and they were more selective regarding the restaurants they went to. They prioritized those that they already knew or those in which they had more confidence or that had outside areas. Figure 3 presents a summary of these barriers and some example transcriptions. On the other hand, age was also an important factor because younger peoplve were impatient to return to restaurants as before the pandemic, whereas older people exhibited insecurity and concern about easing the restrictions.

#### 3.4.3. Behaviors related to food in restaurants

In this section, behaviors related to foods, healthy choices, and takeaway preferences were discussed. Regarding food behavior, when the customers went to a restaurant, they preferred more elaborate dishes over those they could cook in their homes, usually with simple techniques. The customers positively valued offerings of healthy dishes from restaurants, which allowed them to make choices in line with their preferences. Moreover, the customers stated that there was an increase in the number of people interested in local products, minimally processed or unprocessed food, and sustainability. (c) Concerning takeaway food, all participants increased their demand for takeaway food during the pandemic. They believe that over the next year, there will continue to be high demand for takeaway food (d).

- “I believe that now this problematic period is, for restaurateurs, being combined with sustainability, going local, the km 0 movement, all of it. I go to the restaurant and order more elaborate things, but they can be very healthy, and, for example, I prefer that mushrooms come from here and not from I do not know where.”
- “Before, I would already order takeaway food, but as a result of the pandemic, it has become much more accentuated, and I think we will still have another year of high demand for takeaway food.”

#### 3.4.4. Food hygiene and safety

The customers felt comfortable with the measures and precautions that were taken by all staff at the restaurants. They stated that it would be interesting to maintain some of the changes made by restaurants beyond the COVID-19 pandemic, such as the use of a mask during service, as there are other pathogens in circulation, and it would be a way to increase safety. (e) Regarding takeaway food, customers explained that they had never been instructed on how to handle and preserve their food; however, it would be something they would value highly if it were done (f).

- “Yes, I wanted to say that I do not miss anything; in contrast, I believe that there are hygienic things that have come to stay and that are not bad at all because COVID-19 is not the only disease that exists.”
- “No, in my case they have not given me instructions on how to preserve or cook the food, but it would be a nice touch.”

#### 3.4.5. Solutions for restaurateurs provided by customers

The customers explained that some establishments increased their self-service offerings or provided discount vouchers for people who worked nearby. As possible suggestions and solutions, the customers proposed trust and information as key factors needed for them to return to restaurants. They believed that it would be interesting for restaurants to advise and inform customers about the way that they work and about their monitoring of the COVID-19 security measures. (g) They also believed that it was important for restaurants to maintain their essence and take care of the close relationships with customers, waiting times between dishes, the volume of the music, and avoiding placing large groups in the center of the restaurant (h).

- “I think that the key word is information, to give information to the customers, do it well, advise you to do it well and give information, and show us that they do it well.”
- “And I believe that the personal touch that each restaurateur has in their kitchen should not be lost, I mean, they should maintain this because of course, each place has its touch and people go for something that they have and they like. In addition, they cannot lose it.”

### 3.5. Needs and interests of restaurateurs and customers

**Figure 4:**
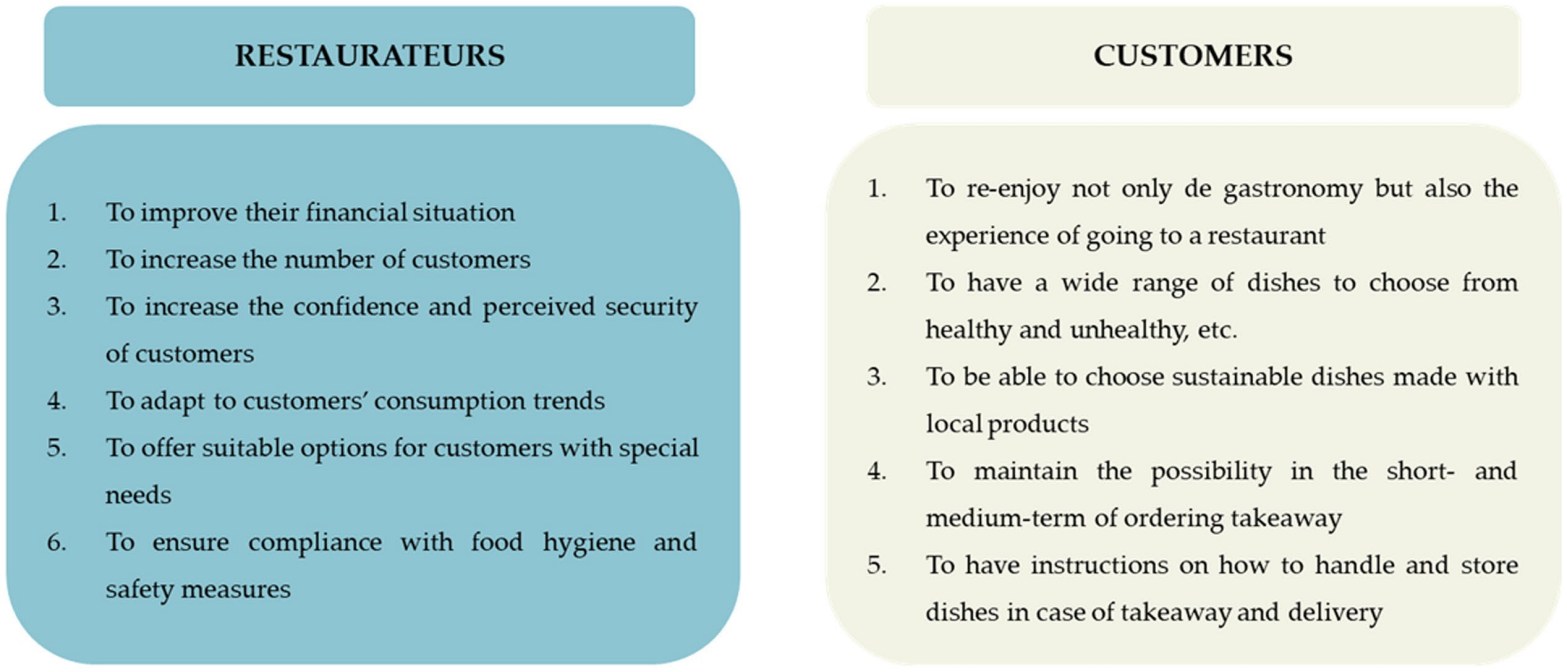
Needs and interests of restaurateurs and customers.

In this results section, different needs and interests were identified through the significant results from the surveys and from the frequency of certain ideas in the focus group discussions with the restaurateurs and customers. Figure 4 presents their needs and interests, including those related to self-security and consumption trends such as local food, among others.

## 4. Discussion

The present cross-sectional study examined the impact of the COVID-19 pandemic on restaurants and their customers through information provided by restaurateurs and customers on a survey and in focus group discussions before and during the current COVID-19 pandemic. This study confirms the hypothesis that the needs, barriers, interests, and food choices of restaurants and customers have changed during the current COVID-19 pandemic.

According to the results, due to the COVID-19 pandemic, customers significantly decreased the frequency with which they went to restaurants while increasing their purchase of takeaway food, and at the same time, restaurants significantly increased their takeaway offerings. Most likely, this may be because of uncertainty, fear, and insecurity related to the pandemic situation, as they commented in the focus group. In this line, a narrative review highlighted a few directions for optimizing the at-home multisensory dining experience due to the takeaway format increasing during and after the pandemic (25).

Moreover, customers preferred to access menus in digital form and pay with cards rather than cash, leading to the need for restaurateurs to adapt. In the same way, restaurants increased their use of digital menus, although currently, only 28 of the 44 total restaurants who answered the survey offer a digital menu. Because restaurant menus are one of the most important types of marketing to promote business, and as we mentioned before, customers prefer menus in digital form, restaurants could improve their menus in digital form with an innovative approach (26).

A 2018 report about the behavioral habits and trends related to restaurants in Catalonia stated that 78% of subjects between 18 and 75 years of age ate outside of their home at least once a week (27). In the present study, even though the data focused only on restaurants in Tarragona Province, which was currently under COVID-19 pandemic restrictions, 43.9% of customers ate outside their home at least once a week, whereas 74.2% before the COVID-19 pandemic, in the same line as the 2018 report.

Regarding restaurant menu information, the results of the present study show that 59.1% of customers now use websites to obtain information. Our results are in accordance with a 2018 report that stated that 35.2% of people, especially young people (48.7%), search for information about restaurants on the Internet (27). Given this new demand, restaurants should consider maintaining updated websites to attract customers.

In the present results regarding Mediterranean offerings, some of the AMed criteria for restaurants, which include the use of olive oil, vegetable dishes, fruit desserts, and local products, among others, were positively perceived by customers. These results are in line with other studies that tried to help restaurateurs better adapt their menus to the Mediterranean diet or healthier diets by giving personalized directions to restaurateurs regarding the dishes listed on their menus. Such instructions included introducing more side vegetables, offering fruit for dessert, and using extra virgin olive oil for different types of cooking (20, 28–30). Additionally, the pandemic resulted in some changes to customer preferences, and restaurateurs could be interested in acting on those changes, such as by offering more vegetarian/vegan or allergen-free dish choices. The fact that our customer respondents increased their demand for local products could indicate an increasing awareness of the importance of sustainability for the planet (31).

In the present study, the results regarding hygiene and food safety, especially as they related to COVID-19, indicate that the restaurateurs increased their use of a hydroalcoholic solution and were much stricter about cleaning and disinfecting the restaurant and surfaces. In addition, the frequency with which restaurateurs cleaned significantly increased by 21.1%. Specifically, the number of restaurateurs who cleaned ≥2 times/day, particularly in the goods reception area, increased, a change highly valued by customers. Moreover, according to the focus groups, restaurateurs and customers were worried and awarded the importance of hygiene and food safety because customers were comfortable with new hygiene and food safety measures implemented by the restaurateurs. Currently, according to the Centers for Disease Control and Prevention (CDC), customers have tips to avoid food poisoning while eating out (32), and restaurants have information in some reports that highlights state food safety practices (33). This information goes in the same line as the results of the present study, highlighting that the present study shows information more specific to the local population. Additionally, in 2020, the guidelines published by the Catalan Food Safety Agency and the Technical Report of the European CDC (34, 35) became much stricter regarding the cleaning of rooms, facilities, surfaces and utensils, offices, furniture, changing rooms, hygienic service areas, and areas of frequent contact with the hands as measures to prevent the spread of SARS-CoV-2. Moreover, the recommended authorized disinfectant products were required to be registered in the Register of Nonagricultural Pesticides or Biocides or in the Official Register of Biocides of the General Directorate of Public Health, Quality and Innovation of the Ministry of Health (34, 35).

However, for general preventive cleaning and disinfection in restaurants, cleaning with water and detergents and the use of common disinfectant products was described as sufficient, but not in situations in which a possible case of COVID-19 was suspected (34, 35). In this context, in the present study, the use of a hydroalcoholic solution for disinfecting cooking utensils increased significantly by 13.7% from the period before to the period during the COVID-19 pandemic, although the most common option is to clean with hot soapy water. This motivation for cleaning is consistent with global recommendations to reduce the number of infections (36).

On the one hand, in the present study, the main barriers facing restaurateurs, detected through the focus groups, were verified, such as the measures imposed by the administration, the county and municipal lockdowns, and the limitations on hours and capacity, inducing a bad economic situation after the start of the COVID-19 pandemic, and emotional consequences such as anxiety or depression among the restaurateurs. One of the main concerns of our restaurateur respondents was their current economic situations, which were difficult because they had many economic losses due to the very restrictive regulations on restaurants that were constantly changing. The main restrictions were the absolute closure of all restaurants, restrictions on hours of operations, the requirement to provide exclusively outdoor seating such as terraces, limits on the numbers of tables and people, and reductions in restaurant capacity, depending on the wave of the pandemic (37).

On the other hand, in line with the focus group, the main barriers experienced by our customer respondents were the restrictions and limitations and the emotional consequences. In the present study, we verified that most of the customers reported a loss of social relationships and freedom after the start of the pandemic. This is justified because in Spain, the cultural practice of eating outside the home is driven by pleasure and social interactions with friends and family (38). In Spain, food is considered a means of socialization between individuals (38), and due to the pandemic, this has been very limited.

Finally, on the basis of the results of surveys and focus groups of the present cross-sectional study, some solutions could be implemented to enhance the COVID-19 consequences, such as reducing costs and offering discounts or reinventing themselves but taking into account customer preferences and comfort. Additionally, based on the needs and interests of restaurateurs and customers, some proposals for how restaurateurs can improve and adapt to new demands include increasing social media and technology in their sector, maintaining and updating hygiene and food security measures, maintaining and increasing AMed criteria according to customers’ preferences, improving takeaway services, and strengthening restaurant essence (Table 5).

**Table 5:** Proposals to improve and adapt to the new demands faced by restaurateurs.

For restaurateurs	
Social media and technology	Promote the use of social media and websites to advertise the restaurant and inform customers about its menu.
	Increase the use of information and communication technology (ICT) for viewing the daily menu and placing orders, and have other alternatives for customers less familiar with ICT.
Hygiene and food security	Review and, if necessary, adapt to the measures recommended by the food safety agency about cleaning the different surfaces of the restaurant.
	Highlight the restaurant’s compliance with the security measures.
	Ensure a safe distance between tables and customers, and prioritize seating on terraces and in well-ventilated areas of the restaurant whenever possible.
	Maintain the use of masks among dining room and kitchen staff and the additional hygiene practices after the COVID-19 pandemic ends.
Mediterranean offerings, customer food preferences	Promote and use local and seasonal products.
	Try to incorporate vegetarian/vegan dishes, allergen-free dishes, and whole grain products, as more than 50% of customers value these options.
	Include and promote dishes adapted to meet special needs and preferences. Obtain advice from professionals if necessary because such offerings increase customer confidence.
	Maintain and strengthen the essence of each restaurant despite the COVID-19 measures; customers go to restaurants not only for the food but also for the experience.
Takeaway and delivery instructions	Incorporate and maintain the demand for takeaway and delivery food and increase offerings of country themed and traditional food.
	Include some handling, cooking, and preservation instructions for takeaway and delivery food.
	Maintain the temperature chain (cold/heat) for delivery dishes.
	Consider the use of sustainable materials for takeaway and delivery.
Environment and the essence of the restaurant	Improve customer comfort by focusing on the service, the food, and the atmosphere of the restaurant.

This study has some limitations. First, this cross-sectional study reflects the COVID-19 situation only in restaurants located in Tarragona Province; however, considering that the COVID-19 restrictions on restaurants have been applied in other geographic areas, the results could be generalizable. Second, the use of self-reported answers to online surveys could make it easy to doubt the quality of the answers. However, the collection of qualitative and quantitative information allows us to obtain more information about the needs and interests of restaurateurs and customers in the current COVID-19 pandemic situation. Third and finally, the restaurateurs and consumers who participated in the focus groups may have felt pressure from their peers, which could have affected their answers.

In contrast, as a strength, this cross-sectional study is the first to assess the impact of COVID-19 on restaurants in Catalonia and Spain. For this reason, it is necessary for restaurateurs to implement the above proposals to adapt to the current situation as a way to reduce the negative impact of the pandemic and for researchers to continue investigating these issues.

## 5. Conclusion

In restaurants, the first COVID-19 lockdown increased takeaway orders, increased sanitation, and improved digital communication with customers, while customers highly valued the use of local foods. Therefore, this study provides valuable information on how to adapt gastronomic offerings during this challenging situation.

## Data availability statement

The raw data supporting the conclusions of this article will be made available by the authors, without undue reservation.

## Ethics statement

The studies involving human participants were reviewed and approved by Ethics Committee of the Pere i Virgili Institute (ref. 056/2021). The patients/participants provided their written informed consent to participate in this study.

## Author contributions

MB-M, JQ, ST, EL, LT, and RS substantial contributions to the conception or design of the work, or the acquisition, analysis, or interpretation of data for the work, drafting the work or revising it critically for important intellectual content, provide approval for publication of the content, and agree to be accountable for all aspects of the work in ensuring that questions related to the accuracy or integrity of any part of the work are appropriately investigated and resolved. All authors contributed to the article and approved the submitted version.

## Funding

The research work presented in this paper is the outcome of a project funded by both institutions under the collaboration framework agreement between the *Diputació de Tarragona* and the Universitat Rovira i Virgili for the period 2020–2023 with the project code 2020/21: *Impacte de la COVID-19 en els establiments de restauració de la Província de Tarragona”: Anàlisis de les necessitats i interessos dels establiments de restauració i dels clients creant solucions per adaptar-se a la situació post-COVID-19 de la Província de Tarragona*. This article in journal has been possible with the support of the Secretaria d’Universitats i Recerca del Departament d’Empresa i Coneixement de la Generalitat de Catalunya, the European Union (UE) and the European Social Fund (ESF) (2022 FI_B2 00011). The authors acknowledge 2021-SGR-00817 project from Agencia de Gestión de Ayudas Universitarias y de Investigación (AGAUR), Generalitat de Catalunya.

## Conflict of interest

The authors declare that the research was conducted in the absence of any commercial or financial relationships that could be construed as a potential conflict of interest.

## Publisher’s note

All claims expressed in this article are solely those of the authors and do not necessarily represent those of their affiliated organizations, or those of the publisher, the editors and the reviewers. Any product that may be evaluated in this article, or claim that may be made by its manufacturer, is not guaranteed or endorsed by the publisher.
