# Supplementary material for: Exploring restaurant and customer needs, barriers, interests, and food choices induced by the COVID-19 pandemic in Tarragona Province (Catalonia, Spain): A cross-sectional study
Source: Front Public Health. 2023 Apr 11;11:1137512. doi: 10.3389/fpubh.2023.1137512 (PMC10126299; doi:10.3389/fpubh.2023.1137512)
Supplement: Supplementary file 2 [file Table_2.doc]

**Supplementary material 2.** COREQ (COnsolidated criteria for REporting Qualitative research) Checklist.

| **Topic** | **ItemNo.** | **Guide Questions/Description** |  |
| --- | --- | --- | --- |
| **Domain 1: Research team and reﬂexivity** | | | |
| *Personal characteristics* | | | |
| Interviewer/facilitator | 1 | Which author/s conducted the interview or focus group? | ST, EL and LT |
| Credentials | 2 | What were the researcher’s credentials? E.g. PhD, MD | SI: PhDs  EL: PhD  LT: PhD |
| Occupation | 3 | What was their occupation at the time of the study? | Researcher |
| Gender | 4 | Was the researcher male or female? | Females |
| Experience and training | 5 | What experience or training did the researcher have? | Researchers in health promotion (including different qualitative and quantitative methods expertise) |
| *Relationship with participants* | | | |
| Relationship established | 6 | Was a relationship established prior to study commencement? | Any |
| Participant knowledge of  the interviewer | 7 | What did the participants know about the researcher? e.g. personal  goals, reasons for doing the research | Goals of the project |
| Interviewer characteristics | 8 | What characteristics were reported about the inter viewer/facilitator?  e.g. Bias, assumptions, reasons and interests in the research topic | Reasons and interests in the research topic |
| **Domain 2: Study design** | | | |
| *Theoretical framework* | | | |
| Methodological orientation and Theory | 9 | What methodological orientation was stated to underpin the study? e.g. grounded theory, discourse analysis, ethnography, phenomenology,  content analysis | content analysis |
| *Participant selection* | | | |
| Sampling | 10 | How were participants selected? e.g. purposive, convenience,  consecutive, snowball | Purposive |
| Method of approach | 11 | How were participants approached? e.g. face-to-face, telephone, mail,  email | Email and telephone |
| Sample size | 12 | How many participants were in the study? | A total of 189 participants were included in the present study: 51 restaurateurs and 138 customers |
| Non-participation | 13 | How many people refused to participate or dropped out? Reasons? | --- |
| *Setting* | | | |
| Setting of data collection | 14 | Where was the data collected? e.g. home, clinic, workplace | Community |
| Presence of non-  participants | 15 | Was anyone else present besides the participants and researchers? | No |
| Description of sample | 16 | What are the important characteristics of the sample? e.g. demographic  data, date | Demographic data |
| *Data collection* | | | |
| Interview guide | 17 | Were questions, prompts, guides provided by the authors? Was it pilot  tested? | Questions were approved by Ethical Committee. |
| Repeat interviews | 18 | Were repeat inter views carried out? If yes, how many? | 3 |
| Audio/visual recording | 19 | Did the research use audio or visual recording to collect the data? | Audio recording |
| Field notes | 20 | Were ﬁeld notes made during and/or after the inter view or focus group? | Yes |
| Duration | 21 | What was the duration of the inter views or focus group? | Around 90 minuts |
| Data saturation | 22 | Was data saturation discussed? | Yes |
| Transcripts returned | 23 | Were transcripts returned to participants for comment and/or | The transcripts no, but the main results yes. |
| Developed from: Tong A, Sainsbury P, Craig J. Consolidated criteria for reporting qualitative research (COREQ): a 32-item checklist for interviews and focus groups. *International Journal for Quality in Health Care*. 2007. Volume 19, Number 6: pp. 349 – 357 | | | |
