# Supplementary material for: Exploring restaurant and customer needs, barriers, interests, and food choices induced by the COVID-19 pandemic in Tarragona Province (Catalonia, Spain): A cross-sectional study
Source: Front Public Health. 2023 Apr 11;11:1137512. doi: 10.3389/fpubh.2023.1137512 (PMC10126299; doi:10.3389/fpubh.2023.1137512)
Supplement: Supplementary file 3 [file Table_3.doc]

**Supplementary material 3.** Frequency and method of cleaning and disinfection for the different areas of the restaurants.

| **Variables** | **Frequency / Method** | **Before COVID n= 44 % (n)** | **Currently COVID^#^ n= 44 % (n)** | **p-value*** |
| --- | --- | --- | --- | --- |
| Goods reception area | ≥2 times/day | 72.1 (31) | 93.2 (41) | 0.022 |
|  | 1 time/day | 20.9 (9) | 6.8 (3) | 0.109 |
|  | <1 times/day | 7.0 (3) | 0.0 (0) | --- |
| Kitchen | ≥2 times/day | 90.9 (40) | 95.0 (38) | 1 |
|  | 1 time/day | 9.1 (4) | 5.0 (2) | 1 |
|  | <1 times/day | 0.0 (0) | 0.0 (0) | --- |
| Counter | ≥2 times/day | 93.2 (41) | 93.2 (41) | 1 |
|  | 1 time/day | 6.8 (3) | 6.8 (3) | 1 |
|  | <1 times/day | 0.0 (0) | 0.0 (0) | --- |
| Takeaway food collection area | ≥2 times/day | 92.5 (37) | 95.0 (38) | 1 |
|  | 1 time/day | 5 (2) | 5.0 (2) | 1 |
|  | <1 times/day | 2.5 (1) | 0.0 (0) | --- |
| Dining room | ≥2 times/day | 95.5 (42) | 95.5 (42) | 1 |
|  | 1 time/day | 4.5 (2) | 4.5 (2) | 1 |
|  | <1 times/day | 0.0 (0) | 0.0 (0) | --- |
| Changing rooms, lockers, and toilet areas | ≥2 times/day | 67.4 (29) | 74.4 (32) | 0.25 |
|  | 1 time/day | 25.6 (11) | 23.3 (10) | 1 |
|  | <1 times/day | 7.0 (3) | 2.3 (1) | 0.5 |
| Cooking utensils | Hot soapy water | 81.8 (36) | 77.3 (34) | 0.625 |
|  | Bleach | 38.6 (17) | 43.2 (19) | 0.625 |
|  | 70% alcoholic solution | 6.8 (3) | 20.5 (9) | 0.031 |
|  | Rinse with water | 99.1 (4) | 99.1 (4) | 1 |
|  | Other products without alcohol or bleach | 20.5 (9) | 15.9 (7) | 0.688 |
| Surfaces | Hot soapy water | 72.7 (32) | 70.5 (31) | 1 |
|  | Bleach | 43.2 (19) | 52.3(23) | 0.219 |
|  | 70% alcoholic solution | 11.4 (5) | 29.5 (13) | 0.08 |
|  | Rinse with water | 4.5 (2) | 6.8 (3) | 1 |
|  | Other products without alcohol or bleach | 13.6 (6) | 22.7 (10) | 0.219 |
| Facilities | Hot soapy water | 59.1 (26) | 63.6 (28) | 0.688 |
|  | Bleach | 56.8 (25) | 61.4 (27) | 0.688 |
|  | 70% alcoholic solution | 15.9 (7) | 25 (11) | 0.219 |
|  | Rinse with water | 4.5 (2) | 6.8 (3) | 1 |
|  | Other products without alcohol or bleach | 9.1 (4) | 18.2 (8) | 0.125 |
| Refrigeration and/or freezing appliances | Hot soapy water | 72.7 (32) | 68.2 (30) | 0.625 |
|  | Bleach | 38.6 (17) | 38.6 (17) | 1 |
|  | 70% alcoholic solution | 9.1 (4) | 20.5 (9) | 0.063 |
|  | Rinse with water | 9.1 (4) | 9.1 (4) | 1 |
|  | Other products without alcohol or bleach | 22.7 (10) | 25 (11) | 1 |
| Cooking and/or heating appliances | Hot soapy water | 77.3 (34) | 72.7 (32) | 0.5 |
|  | Bleach | 22.7 (10) | 25 (11) | 1 |
|  | 70% alcoholic solution | 11.4 (5) | 18.2 (8) | 0.25 |
|  | Rinse with water | 9.1 (4) | 9.1 (4) | 1 |
|  | Other products without alcohol or bleach | 20.5 (9) | 29.5 (13) | 0.219 |

*McNemar test

**^#^** Currently COVID-19 pandemic situation
